# EDEM-based study on the adjustable feeding parameters of square bale maize straw bale-breaking device

**DOI:** 10.1371/journal.pone.0317838

**Published:** 2025-01-30

**Authors:** Tiejun Wang, Tinghe Zhang, Shuai Yu, Hongguang Cui, Ruili Wang

**Affiliations:** College of Engineering, Shenyang Agricultural University, Shenyang, Liaoning Province, People’s Republic of China; Sakarya Uygulamali Bilimler Universitesi, TÜRKIYE

## Abstract

One of the primary challenges faced by small rubbing filament machines is the significant variability in material sizes, particularly in the feeding direction. This variability complicates the processing of locally baled straw with a single device. To address this issue, an adjustable feeding and bale-breaking device was developed and tested to enhance the filamentous performance of baled straw. The machine integrates a series of bale-breaking knives along with a pair of feeding and bale-breaking rollers. This paper presents an overview of the machine’s structure and operating principles, alongside an analysis of the forces acting on the straw within the device, which informed the design of key components and devices. A discrete element simulation model suitable for square baled-straw has been established, providing a research foundation for the subsequent optimization of device design parameters. Effects of motor bale-breaking roller rotating speed (*x*_1_), bale-breaking roller height (*x*_2_) and bale-breaking knife quantity (*x*_3_) on the productivity of bonding bond destruction rate (*Y*_1_) and the particle average speed (*Y*_2_) were explored. Three-dimensional quadratic regression orthogonal rotation central combination experiment method combined with response surface method was used to conduct experiments and explore the interaction effects of influence factors on indicators. A regression model of influence factors and evaluation indicators was established through the analysis of variance. The significant factors affecting *Y*_1_ were ordered of *x*_1_, *x*_2_, *x*_3_, and the significant factors affecting *Y*_2_ were ordered of *x*_2_, *x*_3_, *x*_1_. In the interaction of factors, *x*_1_*x*_2_ *and x*_2_*x*_3_ had an extremely significant impact, and *x*_1_*x*_3_ had a significant impact on *Y*_1_; *x*_1_*x*_2_, *x*_1_*x*_3_ and *x*_2_*x*_3_ had a significant impact on *Y*_2_. The optimal structure and working parameters combination were determined to be 1448 rpm for *x*_1_, 268 mm for *x*_2_, and 14 pieces for *x*_3_. Verification experiments demonstrated that the actual values were 96.95% for the straw rubbing rate and 235.13 kg/(kW·h) for the per unit power productivity. The operation of the adjustable feeding and bale-breaking device developed in this study proved effective in enhancing productivity and breaking performance during the feeding of baled straw. It successfully met the design requirements for the grain size necessary for the comprehensive utilization of straw. Overall, this research establishes a foundational basis for the further development of a small, multipurpose straw rubbing filament machine.

## Introduction

In China, the annual production of maize straw exceeds 2.6×10^11^ kg. As a recycling method, part of these straw is typically compressed into square-baled maize straw [1], which increase the bulk density of the maize straw, reduces storage space, and thereby mitigates agricultural ecology environmental pollution [2–4]. However, the utilization of untreated square-bale maize straw in Northeast China is restricted by long-term low temperature after the harvest season [5]. Therefore, promoting the mechanical treatment and utilization of square-baled maize straw has become an important issue [6]. Currently, the main method of bale-breaking is still manual in China, which is labor-intensive, time-consuming, and costly approach [5]. Therefore, it is necessary to find an automated mechanical treatment solution. The fixed feeding device of the existing maize straw rubbing filament machine, which is suitable for the whole-plant maize straw crushing and kneading operation, hinders the smooth feeding of square-baled maize straw and reduces the production efficiency. It is not only difficult to deal with baled straw, but also unable to meet the different external dimensions of square-baled maize straw. Because the baling machines have differences in the working principle and operating parameters, making the outer size of baled straw varies greatly. Referring to Rectangular bale baler [7], rectangular square-baled straw with a size below 500mm was generally small, 500~800mm was medium, and greater than 800mm was large. However, the overall size of the whole-plant stalk is generally 150~260mm. Therefore, it is urgent to find an adjustable feed feeding operating system.

Straw is a biological material with anisotropic and viscoelastic complex structure [8, 9], especially among the square-bale maize straw. With proper design and structure parameters, the feeding and breaking system usually consists of a pair of rollers installed in the same vertical direction to clamping and breaking the straw at a desired level. Several types of bale-breaking device have been developed over the past few decades. A typical processor for baled crop material comprising a container, a disintegrator, a discharge opening and means for; manipulating the crop material for disintegration by the disintegrator [10]. A bale processor, Tomahawk, was first manufactured in 1983 by *Teagle* machinery [11]. Mao et al. [12] developed a bale loosing and crude shredding machine, which used the combination of fly-wheel and hammer chopped mode achieved the functions of bale loosing and crude shredding to some extent. Igathinathane et al. [13] evaluated the cutting orientation effects of maize straw based on the experiment analysis of cutting force-deformation characteristics. A roselle harvester which consisted of two pair of geared rollers with *Nylamid* teeth has been developed and tested [14]. Zhang et al. [15] simulated a process involved the kneading and crushing of maize straw model, the parameters calibration for the bonded particle contact model of maize straw were determined and carried out based on the discrete element model for bimodal distribution, which could provide basis and guidance for theoretical calculation.

To enhance the understanding and quantification of the performance of the square-bale maize straw feeding and breaking system, a square-bale maize straw adjustable feeding and bale-breaking device was developed in this study. The objective was to establish a comprehensive set of evaluation criteria to assess the impact of key design and mechanical parameters on the performance of the square-bale maize straw adjustable feeding and bale-breaking system. Specifically, the study focused on the effects of the bale-breaking roller’s rotational speed, roller height, and the number of bale-breaking knives on the bonding-bond destruction rate and particle average speed. A feeding and breaking simulation model was developed, followed by optimization experiments and response surface analysis. The findings of this study are anticipated to provide valuable guidelines for the parameter configurations of the baled maize straw filamentous machine.

## Materials and methods

### Adjustable feeding device and square-bale maize straw model establishment

**Fig 1:**
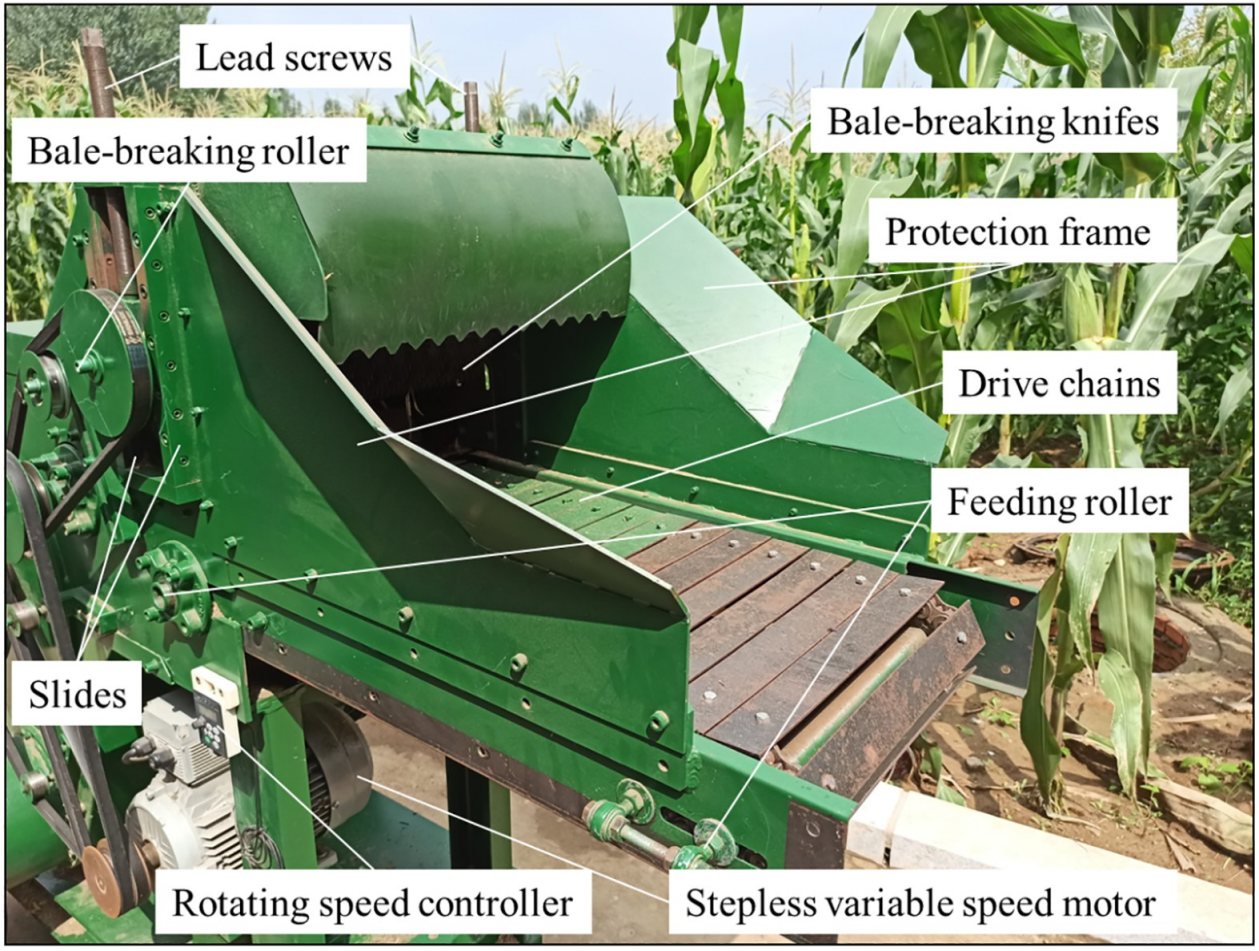
Diagram of adjustable feeding and bale-breaking device.

The machine structure was shown in Fig 1, the adjustable feeding and breaking system developed in this study mainly consisted of a group of bale-breaking knifes and a pair of feeding and bale-breaking rollers. The diameter of the feeding roller and bale-breaking roller was 450 mm, and the surface of the bale-breaking roller was covered with sleeves to limit the position of the bale-breaking knife. A group of feeding rollers was linked with drive chains, and the bale-breaking roller was connected by transmission belt. The driving feeding roller and the bale-breaking roller were mounted on the slide through lead screws so that the feeding distance of the driving feeding roller and the bale-breaking roller could be automatically adjusted according to the different diameters of square-bale maize straw. The feeding rollers, bale-breaking roller and other rollers of this square-bale maize straw rubbing machine were driven by one steeples variable speed motor only. According to previous studies [16, 17], a group of bale-breaking knifes made of 65 Mn and YG8 tungsten-cobalt carbide material (with a diameter of 180 mm, a number of 40 teeth and a thickness of 2.2 mm) were mounted evenly on the bale-breaking roller shaft.

**Fig 2:**
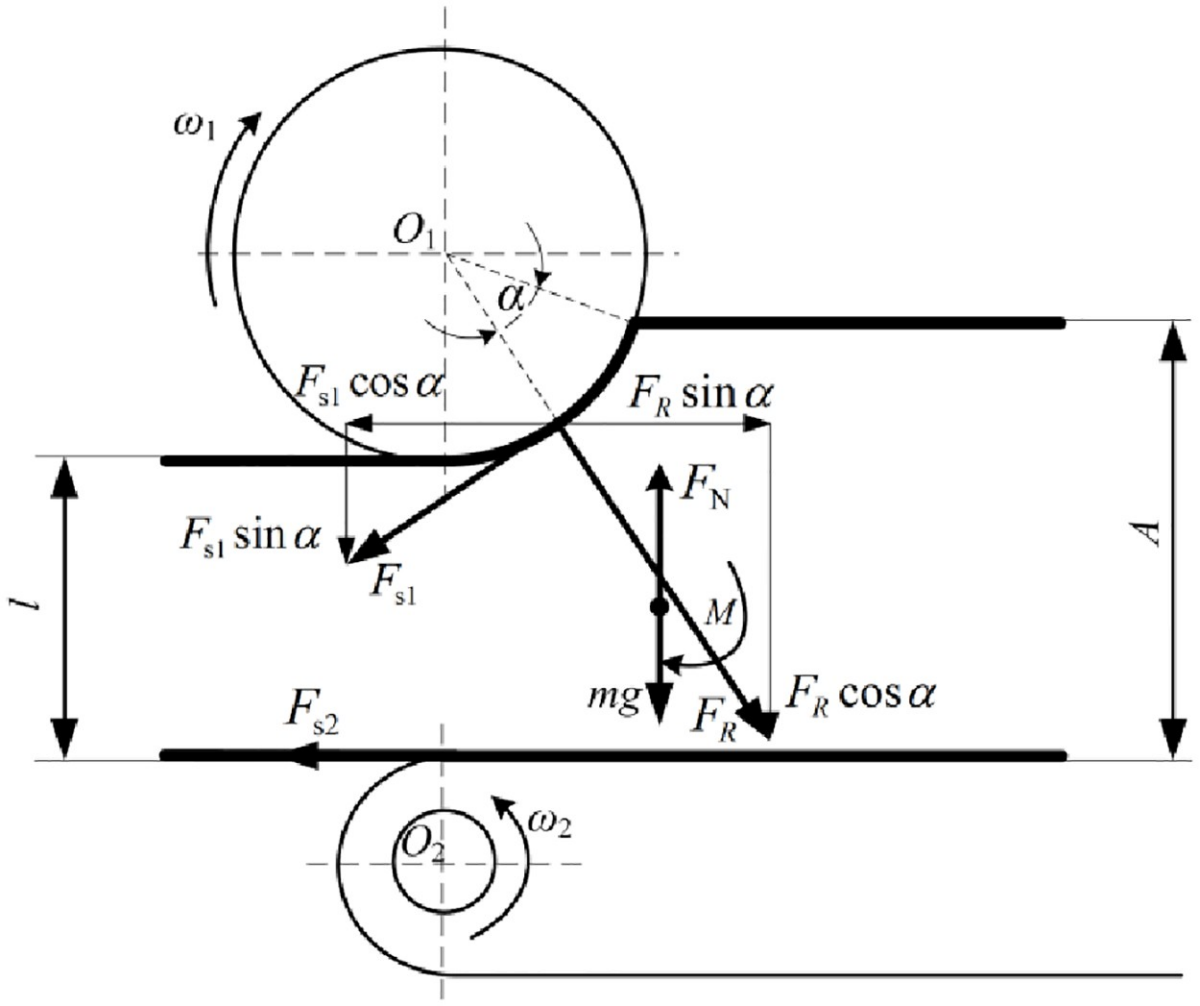
Analysis of force and movement of the straw bale-breaking and feeding process.

The machine principle was shown in Fig 2, saw tooth knife head elements fixed to the bale-breaking roller shaft were jagged, and the angle of each rake and relief saw tooth was 0.5 °, which restricted the circumferential movement of straws and ensured smooth conveying. To explore the influence of the distance between the driving feeding roller and the bale-breaking roller element on bale-breaking performance, the height adjustment mechanism of the bale-breaking roller were combined of lead screw and slide rail, and the feeding distance could be adjusted using the screw nuts on the lead screw and slide rail. In bale-breaking process, the square-bale maize straw was fed into the feeding port from the smallest surface and conveyed backwards to the shredding system under the friction of the driving feeding roller and the bale-breaking roller. The driving feeding roller supported the maize straw and the bale-breaking roller elements struck the moving maize straw at a high frequency as the bale-breaking roller rotated. Thus, with appropriate feeding roller speed, bale-breaking roller rotating speed, feeding height between driving feeding roller and bale-breaking roller elements and quantity of bale-breaking knife, square-bale maize straw could be broken by the bale-breaking knife at the required level. The driving feeding roller speed and bale-breaking roller rotating speed mainly affected the striking frequency of bale-breaking elements on square-bale maize straw, the quantity of bale-breaking knife and bale-breaking roller rotating speed affected the impact strength, and the feeding height between driving feeding roller and bale-breaking roller elements mainly affected the impact angle and the interaction area of bale-breaking knife on square-bale maize straw.

As shown in Fig 2, the vertical component of tangential force (***F***_***N*1**_) of the bale-breaking knife was the main impact force for bale-breaking, and the maize straw could be conveyed backwards by ***F***_***f*1**_ and ***F***_***f2***_. Assuming that the square-bale maize straw is cuboid, and the square-bale maize straw passes through the middle of the driving feeding roller and bale-breaking roller. Thus, the necessary conditions for the bale-breaking roller to ensure the square-bale maize straw feeding are:

$$F_{f1}\;\text{cos}\;\alpha +F_{f2}⩾F_{N1}\;\text{sin}\;\alpha$$ (1)

α—Feeding angle, °;

***F***_***N*1**_—The supporting force of the feeding roller on the square-bale maize straw, N;

***F***_***f*1**_—The friction force of the bale-breaking knife on the square-bale maize straw, N;

***F***_***f*2**_—The friction force of the drive chains on the square-bale maize straw, N.

It can be calculated from the geometric relationship:

$$F_{N2}=F_{f1}\;\text{sin}\;\alpha +F_{N1}\;\text{cos}\;\alpha +mg$$ (2)

$$F_{f1}=f_{1}\cdot F_{N1}$$ (3)

$$F_{f2}=f_{2}\cdot F_{N2}$$ (4)

*m*—Square-bale maize straw quality, kg;

***F***_***N*2**_—The positive pressure of the bale-breaking knife on the square-bale maize straw, N;

*f*_1_—The friction factor between the bale-breaking knife and the square-bale maize straw;

*f*_2_—The friction factor between the drive chains and the square-bale maize straw.

Assuming that the material of the bale-breaking knife and the drive chain are the same, the friction factor of the square-bale maize straw and the two are the same, that is, *f*_1_ = *f*_2_ = *f*, and the combination of (1)–(4) gives

$$2f\;\text{cos}\;\alpha +f^{2}\;\text{sin}\;\alpha \text{+}\frac{f\cdot mg}{F_{N1}}⩾\;\text{sin}\;\alpha$$ (5)

Where

$$f^{2}\;\text{sin}\;\alpha \text{+}\frac{f\cdot mg}{F_{N1}}⩾0$$ (6)

Then the formula (1) is established and only needs to be satisfied:

2fcosα≥sinα (7)

The feeding angle α can be expressed as follows:

2tanφ≥tanα (8)

Generally, the value range of straw friction angle *φ* is 17°~27° [18], and the value range of *α* is 32°~46°. The size of the feeding angle is affected by the feeding gap and the height of the square-bale maize straw. Therefore, when the height of the square-bale maize straw is fixed, the size of the feeding gap directly affects the size of the feeding angle. In order to further clarify the effect of the feeding gap on the square-bale maize straw of feeding performance and bale-breaking effect needs to be optimized by experiment and simulation.

For the square-bale maize straw feeding stable, the following boundary condition must be met:

A−l=r−rcos2α (9)

*l*—The feeding gap between feeding roller and bale-breaking roller elements, mm;

*r*—The radius of bale-breaking knife, mm;

*A*—The height of the square-bale maize straw, mm.

In this study, *A* = 360 mm, *r* = 90 mm, and *α* = [31.8 °, 45.6 °]. To break square-bale maize straw effectively, the feeding gap between driving feeding roller and bale-breaking roller elements (*l*) needed to be 268 mm to 310 mm.

### Materials

The accuracy and reliability of the developed simulation model is the basis for the correct implementation of the overall simulation experiment, while proper simplification of the model can greatly reduce the simulation difficulty. The following simplifications are thus made in the present simulation [19].

1. The difference between the epidermis and inner core of maize straws is ignored and they are regarded as one and replaced by materials with physical parameters similar to those of the whole-plant maize straw in the simulation.
2. The negligible effect of leaves is neglected in the finite element simulation analysis.
3. It is assumed that the frictional coefficient of the maize straw does not change with deformation of the structure.

**Fig 3:**
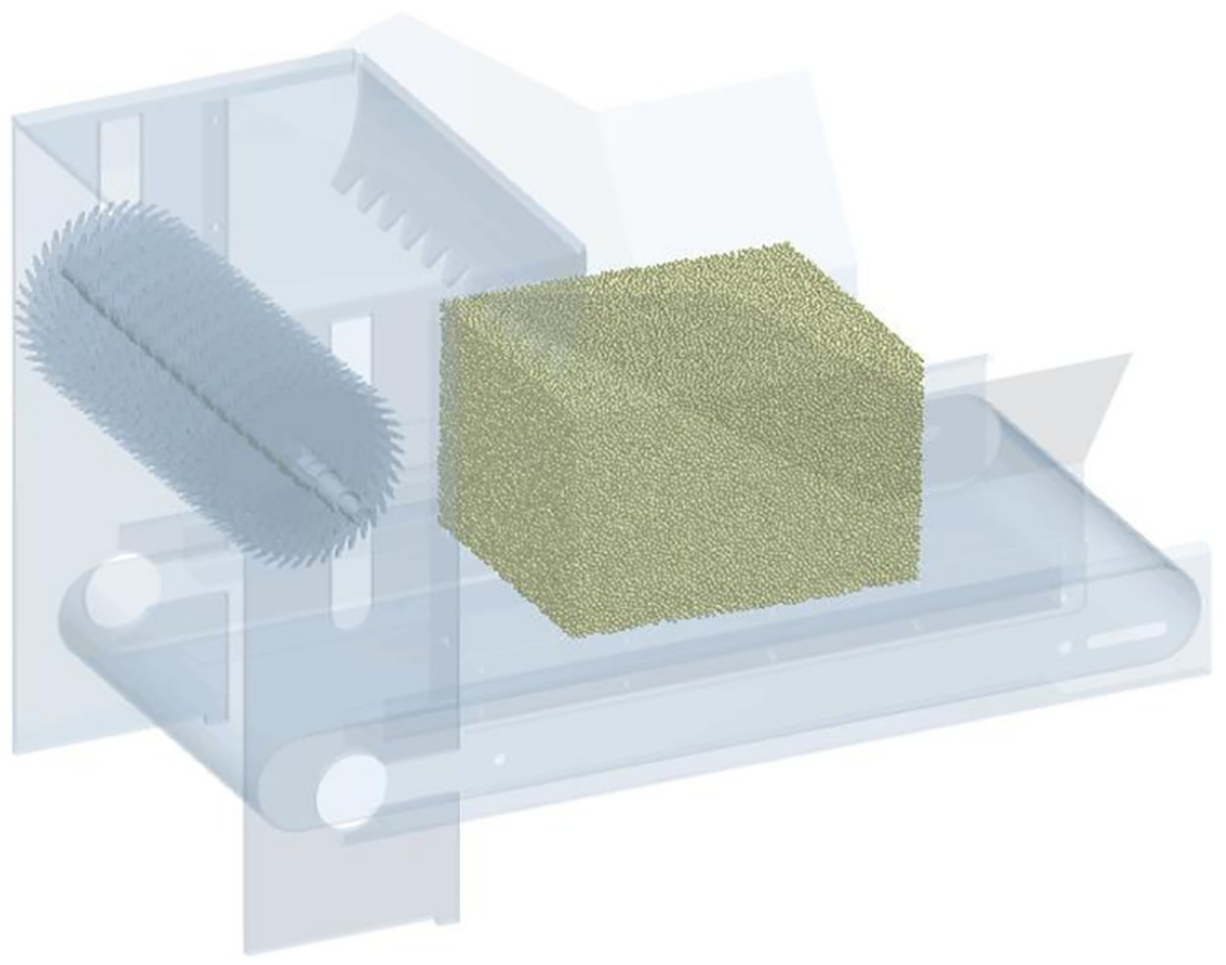
Model of feeding and breaking device and square-bale maize straw.

Based on the optimal structure and operating parameters obtained above, a three-dimensional simplified square-bale maize straw adjustable feeding and breaking simulation model was created, which consisted of a pair of feeding rollers and a bale-breaking roller with a group of bale-breaking knifes (as shown in Fig 3). The square-bale maize straw adjustable feeding and breaking model was imported into EDEM dynamic analysis software. The kinematic pairs of each component were determined, and constraints and drives were added. The dynamic simulation analysis was finally performed in EDEM software.

### Dynamic simulation

The feeding and bale-breaking rigid-flexible coupling model was established using SolidWorks software (SolidWorks Corp., Waltham, MA, USA) and EDEM dynamic analysis software [20]. The following simplifications are thus made in the present simulation.

1. Assuming the maize straw is modeled as a rigid, homogeneous material with specific mechanical properties. Straw is composed of fibrous materials that can be assumed to have uniform properties in a small-scale simulation. This simplification is common in early-stage modeling to facilitate computational efficiency [21].
2. Assuming the interaction between straw particles is modeled using the Hertz-Mindlin model. The Hertz-Mindlin model is suitable for granular materials where contact stiffness, friction, and restitution coefficients can be defined. This model is often validated for agricultural applications, as it effectively captures the behavior of straw particles under compression and impact. Values for static and dynamic friction can be derived from experimental data or literature, ensuring that they represent realistic conditions [22].
3. Assuming the fixed or moving boundary conditions are established based on the physical constraints of the crushing equipment. Defining boundaries reflects the actual design of crushing machinery, ensuring that the simulation accurately represents operational conditions. No-slip Boundary Condition: This is a common assumption for machinery simulations where the material does not slip against the boundary surfaces [23].
4. The particle size distribution of straw is modeled as uniform distribution. Simplifying the particle size distribution allows for manageable computational demands while still capturing the essence of straw behavior during crushing [24].
5. A specific time step is chosen to ensure numerical stability and convergence of the simulation. The time step should be small enough to capture dynamic events of impacts during crushing accurately. A common approach is to perform a sensitivity analysis to validate that the chosen time step does not significantly affect the results [25].

Components of the three-dimensional simulation model in EDEM software were defined as rigid bodies, and attributes of the components were modified. The adjustable feeding and breaking were defined as ordinary steel material with density of 7.8 × 10^3^ kg/m^3^, shear modulus, 7 × 10^10^ Pa and Poisson’s rate 0.3. Material properties of the square-bale maize straw model are shown in Table 1, which were taken from previous studies [26]. Alternate set physical and virtual material properties of the square-bale maize straw model to generate a bonding-bond. The numbers of particles and bonding-bonds after simulation operation were 214,934 and 794,769, respectively. The average number of bonding-bonds around each particle was 3.68, which can provide sufficient bonding force for the square-bale maize straw model.

**Table 1:** Simulation parameters of the feeding device.

Parameters of the square-bale maize straw	Value
Density (kg m^-3^)	1×10^3^
Poisson’s ratio	0.25
Shear modulus (Pa)	1×10^6^
Recovery coefficient between materials	0.2
Static friction coefficient between materials	0.7
Rolling friction coefficient between materials	0.14

### Experimental design

The Box-Behnken design (BBD) is a commonly utilized experimental approach for developing second-order response surface models in optimization research. Serving as an efficient alternative to the full factorial design, BBD allows for the collection of more comprehensive data with a reduced number of experimental trials [27, 28]. In this study, BBD with three factors and three levels was applied to investigate the effects of influence factors on square-bale maize straw feeding and breaking performance. Based on the previous research foundation and relevant research conclusions [29–31], the three investigated factors were the bale-breaking roller rotating speed (*X*_1_), roller height (*X*_2_) and knife quantity (*X*_3_). Levels and codes of these experimental variables are shown in Table 2.

**Table 2:** Levels and codes of experimental variables.

Factors	Symbol		Levels		
	Uncoded	Coded	-1	0	+1
Bale-breaking roller rotating speed (rpm)	*X*_1_	*x*_1_	950	1199	1448
Bale-breaking roller height (mm)	*X*_2_	*x*_2_	268	289	310
Bale-breaking knife quantity (piece)	*X*_3_	*x*_3_	8	11	14

Baled maize straw is a relatively compact assembly of straw with a regular external shape, formed under pressure, which need to be disassembled. In the bale-breaking process, the proportion of successfully maize-broken within unit time is taken as the main assessment criterion with a high proportion displaying good performance. Therefore, in this study, bonding-bond destruction rate (*Y*_1_) and particle average speed (*Y*_2_) were chosen as the experimental indicators. *Y*_1_ and *Y*_2_ were received from EDEM software.

### Data analysis

The experimental data were analyzed using Design-Expert (Stat-Ease, Inc., Minneapolis, MN, USA). Analysis of variance (ANOVA) and significance tests were applied to check the accuracy of the mathematical models. Response Surface Methodology (RSM) is a vital statistical technique employed to model and assess response variables influenced by numerous input factors. This method is effective for developing models that encompass second-order polynomial and higher-degree parameters, rendering it suitable for investigating the interaction effects of variables on the responses [32]. RSM, in conjunction with BBD, is a widely utilized approach for process enhancement, assessment, and optimization. Utilizing results obtained from BBD and subsequent RSM analysis, one can construct second-order polynomial equations comprising linear, quadratic, and interaction terms to depict the relationship between dependent responses and independent variables, expressed as follows [33, 34]:

$$Y=\beta_{0}+\sum_{i=1}^{k}\beta_{i}X_{i}+\sum_{j=1}^{k}\beta_{ii}X_{j}^{2}+\sum_{i=1}^{k}\beta_{ij}X_{ij}+\varepsilon$$ (10)

where, *β*_0_ as the constant; *β*_i_ as item code, examine factors; *β*_ii_ in order to investigate the quadratic term of factors coding; *β*_ij_ item in order to investigate the interaction of factors coding *X*_i_; *X*_j_ as independent variables, *ε* for random error.

In this investigation, the statistical significance of the regression coefficients was evaluated using their corresponding p-values, while the model’s adequacy was assessed by the coefficient of determination (*R*^2^). An F-test was employed to confirm the statistical significance of the model. Three-dimensional response surface graphs were generated in Design-Expert based on the equations derived for each response, illustrating the impact of independent variables on the output responses. The optimal values for the selected independent factors were identified by resolving the regression equations using a comprehensive evaluation method.

## Results and discussions

### Analysis of simulation results

**Fig 4:**
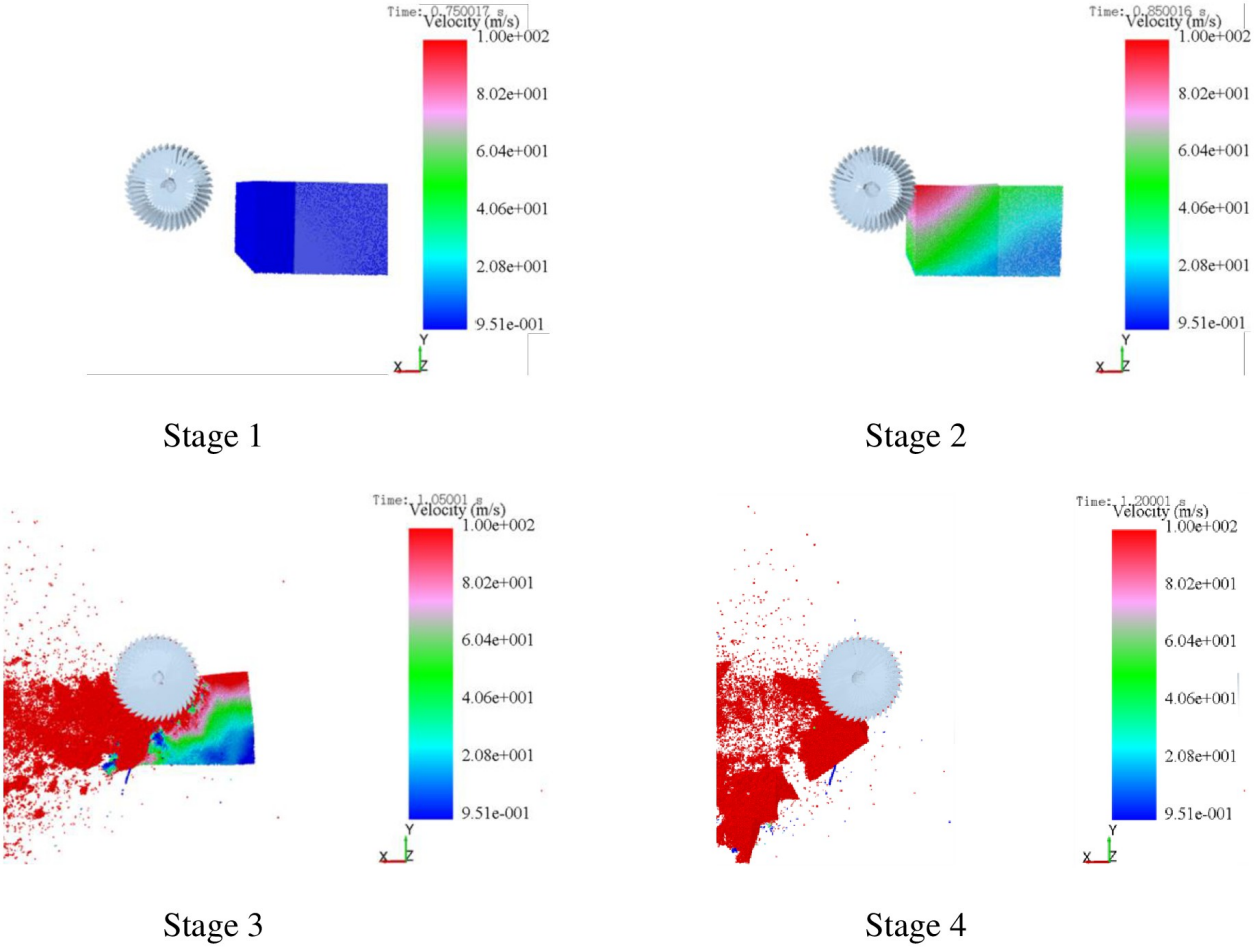
Four stages of feeding square-bale maize straw.

The square-bale maize straw adjustable feeding and breaking processes are shown in Fig 4, which are divided into 4 stages under the optimal operating conditions. The square-bale maize straw moved at a constant speed in the initial phase (Stage 1). In the second stage (Stage 2), when the square-bale maize straw contacted with the rotating bale-breaking knife, the speed of the contact point increased rapidly, and the rear of the square-bale maize straw was temporarily reduced due to the momentary contacted resistance of the bale-breaking roller. Within the feeding progresses (Stage 3), the bale-breaking knife and the chain plate had a continuous clamping effect on the passing square-bale maize straw, whose front part was torn and broken into blocks and rolled back quickly. The square-bale maize straw was equivalent to a cantilever beam at the feeding process, which was conducive to increasing the bale-breaking rate. Stage 4 indicated that the square-bale maize straw was stably conveyed when the speed of the broken particles was close to 100 m/s.

**Fig 5:**
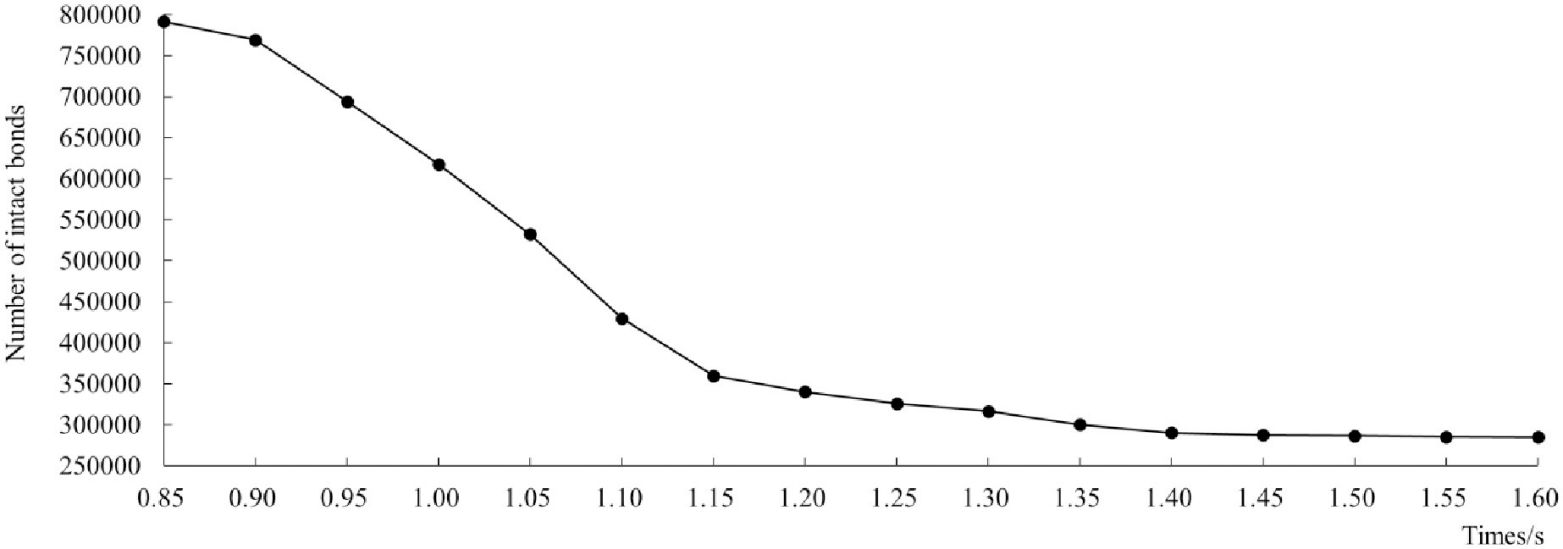
Change curve of bonding-bond corresponding to the four stages of feeding.

The change in the number of the square-bale maize straw bonding-bonds over time was shown in Fig 5. At the beginning (0.00–0.80 s), the square-bale maize straw model was generated in the computational domain generated 791,769 bonding-bonds. Subsequently at 0.80–0.85 s, the number of bonding-bonds was slightly lost as a result of the vibration of the chain plate during the feeding transportation process. In the period of 0.85–1.15 s, the number of bonding-bonds dropped sharply, indicating that the square-bale maize straw began to be severely impacted and broken by the bale-breaking knife, and the computing domain began to be filled with broken particles. At the end (1.15–1.60 s), the overall number of bonding-bonds tends to be stable while the feeding and breaking process of the square-bale maize straw was completed, even though the broken particles fell on the edge of the calculation domain causing a small amount the bonding-bonds to be broken. Therefore, it can be said that the simulation model was reliable.

### Simulation experimental results

Using BBD, 17 experiments with 5 center points were carried out in random order to minimize the effect of any unexplained variability in the observed responses due to external factors. Experiments at each level were repeated 3 times. The BBD matrix and responses are shown in Table 3.

**Table 3:** Factors and responses of experiments.

Trail	Factors			Responses	
	*x*_1_ (rpm)	*x*_2_ (mm)	*x*_3_ (pieces)	*Y*_1_ (%)	*Y*_2_ (m s^-1^)
1	950 (-1)	268 (-1)	11 (0)	52.80	4.24
2	1448 (1)	268 (-1)	11 (0)	60.67	4.29
3	950 (-1)	310 (1)	11 (0)	33.02	3.25
4	1448 (1)	310 (1)	11 (0)	46.89	3.71
5	950 (-1)	289 (0)	8 (-1)	39.51	3.40
6	1448 (1)	289 (0)	8 (-1)	49.98	3.86
7	950 (-1)	289 (0)	14 (1)	45.31	3.97
8	1448 (1)	289 (0)	14 (1)	58.87	4.02
9	1199 (0)	268 (-1)	8 (-1)	52.23	4.26
10	1199 (0)	310 (1)	8 (-1)	38.61	3.15
11	1199 (0)	268 (-1)	14 (1)	61.89	4.23
12	1199 (0)	310 (1)	14 (1)	42.95	3.69
13	1199 (0)	289 (0)	11 (0)	52.11	3.19
14	1199 (0)	289 (0)	11 (0)	53.06	3.31
15	1199 (0)	289 (0)	11 (0)	52.33	3.32
16	1199 (0)	289 (0)	11 (0)	53.05	3.36
17	1199 (0)	289 (0)	11 (0)	52.58	3.47

ANOVA results for the models of *Y*_1_ and *Y*_2_ are shown in Table 4. Results revealed high significance of the regression models (*p* < 0.0100, *p*-values are reported as four decimal places throughout the article). For model term of *Y*_1_, the linear terms *x*_1_, *x*_2_ and *x*_3_, the interaction terms *x*_1_*x*_2_ and *x*_2_*x*_3_, the quadratic terms $x_{1}^{2}$, $x_{2}^{2}$ and $x_{3}^{2}$ were highly significant (*p* < 0.0100), moreover, the interaction terms *x*_1_*x*_3_ was significant (*p* < 0.0500). The *p*-value, determination coefficient *R*^2^ and variation coefficient *CV* of the model were < 0.0001, 99.84% and 0.95, respectively, which indicated significance of the regression model and reliability of the experiment results (*R*^2^ and variation coefficient *CV* are both reported as two decimal places throughout the article). The *p*-value of 0.3331 for lack of fit implied its insignificance comparing to the pure error, indicating that the regression model was effective. For model term of *Y*_2_, the linear terms *x*_1_, *x*_2_ and *x*_3_, the quadratic terms $x_{1}^{2}$, $x_{2}^{2}$ and $x_{3}^{2}$ were highly significant (*p* < 0.0100), moreover, the interaction terms *x*_1_*x*_2_, *x*_1_*x*_3_ and *x*_2_*x*_3_ were significant (*p* < 0.0500). The same statistical analysis for model terms of *Y*_2_ showed that the *p*-values of models was < 0.0001 and the *p*-values of lack of fit was 0.8748, which manifests high significance for the regression models and in significance for the terms of lack of fit. The determination coefficient *R*^2^ and variation coefficient *CV* were 98.28% and 2.23, indicating that the *Y*_2_ model fitted well.

**Table 4:** ANOVA results for experimental data.

Source	df	SS	MS	*F*-value	*p*-value
*Bonding bond destruction rate* (*Y*_1_)					
Model	988.81	9	109.87	491.69	< 0.0001**
*x*_1_	261.86	1	261.86	1171.91	< 0.0001**
*x*_2_	546.48	1	546.48	2445.66	< 0.0001**
*x*_3_	102.89	1	102.89	460.46	< 0.0001**
*x*_1_ *x*_2_	9	1	9	40.28	0.0004**
*x*_1_ *x*_3_	2.39	1	2.39	10.68	0.0137*
*x*_2_ *x*_3_	7.08	1	7.08	31.67	0.0008**
$x_{1}^{2}$	24.09	1	24.09	107.79	< 0.0001**
$x_{2}^{2}$	15.03	1	15.03	67.26	< 0.0001**
$x_{3}^{2}$	13.9	1	13.9	62.19	< 0.0001**
Residual	1.56	7	0.2234		
Lack of fit	0.84	3	0.28	1.55	0.3331
Pure error	0.7241	4	0.181		
Total	990.37	16			
*R*^2^ = 99.84%; adjusted *R*^2^ = 99.64%; *CV* = 0.95.					
Particle average speed (*Y*_2_)					
Model	2.71	9	0.3007	44.36	< 0.0001**
*x*_1_	0.1301	1	0.1301	19.19	0.0032**
*x*_2_	1.3	1	1.3	191.2	< 0.0001**
*x*_3_	0.1922	1	0.1922	28.35	0.0011**
*x*_1_ *x*_2_	0.042	1	0.042	6.2	0.0416*
*x*_1_ *x*_3_	0.042	1	0.042	6.2	0.0416*
*x*_2_ *x*_3_	0.0812	1	0.0812	11.98	0.0105*
$x_{1}^{2}$	0.2874	1	0.2874	42.39	0.0003**
$x_{2}^{2}$	0.3331	1	0.3331	49.13	0.0002**
$x_{3}^{2}$	0.2061	1	0.2061	30.41	0.0009**
Residual	0.0475	7	0.0068		
Lack of fit	0.0068	3	0.0023	0.225	0.8748
Pure error	0.0406	4	0.0102		
Total	2.75	16			
*R*^2^ = 98.28%; adjusted *R*^2^ = 96.06%; *CV* = 2.23.					

^1^ Asterisks indicate significant difference at the (*) 95% and (**) 99% confidence levels.

With insignificant items excluded, the regression equations for each response with independent variables were as follows:

$$\begin{array}{ll} {\hat{Y}}_{1} & =52.63+5.72x_{1}-8.26x_{2}+3.59x_{3}+1.50X_{1}X_{2}\\ & +0.77X_{1}X_{3}-1.33X_{2}X_{3}-2.39X_{1}^{2}-1.89X_{2}^{2}-1.82X_{3}^{2} \end{array}$$ (11)

$$\begin{array}{ll} {\hat{Y}}_{2} & =3.33+0.13X_{1}-0.40X_{2}+0.16X_{3}+0.10X_{1}X_{2}\\ & -0.10X_{1}X_{3}+0.14X_{2}X_{3}+0.26X_{1}^{2}+0.28X_{2}^{2}+0.22X_{3}^{2} \end{array}$$ (12)

As shown in Table 4, the impact (from high to low) of bale-breaking roller rotating speed (*x*_1_), bale-breaking roller height (*x*_2_), and bale-breaking knife quantity (*x*_3_) on the bonding-bond destruction rate is ordered as *x*_2_ > *x*_1_ > *x*_3_ based on *F* values, and the interactive impact of combined factors is ordered as *x*_1_*x*_2_ > *x*_2_*x*_3_ > *x*_1_*x*_3_. Based on the *F* values in Table 4, bale-breaking roller height (*x*_2_) was ranked as the first factor that determined the particle average speed, and this is followed by bale-breaking knife quantity (*x*_3_) and bale-breaking roller rotating speed (*x*_1_). The order of the interactive impact of combined factors was *x*_2_*x*_3_ > *x*_1_*x*_3_ > *x*_1_*x*_2_. Generally, the influence of the experimental factor *x*_1_, *x*_2_ and *x*_3_ on the two experimental indexes are extremely significant.

The findings regarding the significant influence of the feed roller speed, feed gap, and knife quantity in the operation of straw bale-breaking and rubbing filament machines represent a novel contribution to the design and optimization of baled straw bale-breaking and rubbing filament machine in agricultural machinery. These parameters have traditionally been recognized as important in machine performance [35–39], but their specific effects, particularly in the context of square-baled straw bale-breaking and rubbing filament systems, have not been as deeply explored or quantified in previous studies. While the importance of these parameters was known in theory, the research takes a significant step by providing quantitative analysis and developing models that define how changes in feed roller speed, feed gap, and knife quantity influence key performance indicators. This quantification enables engineers and machine designers to create more efficient, customizable, and adaptable straw treatment machines.

### Interactive analysis and discussion

**Fig 6:**
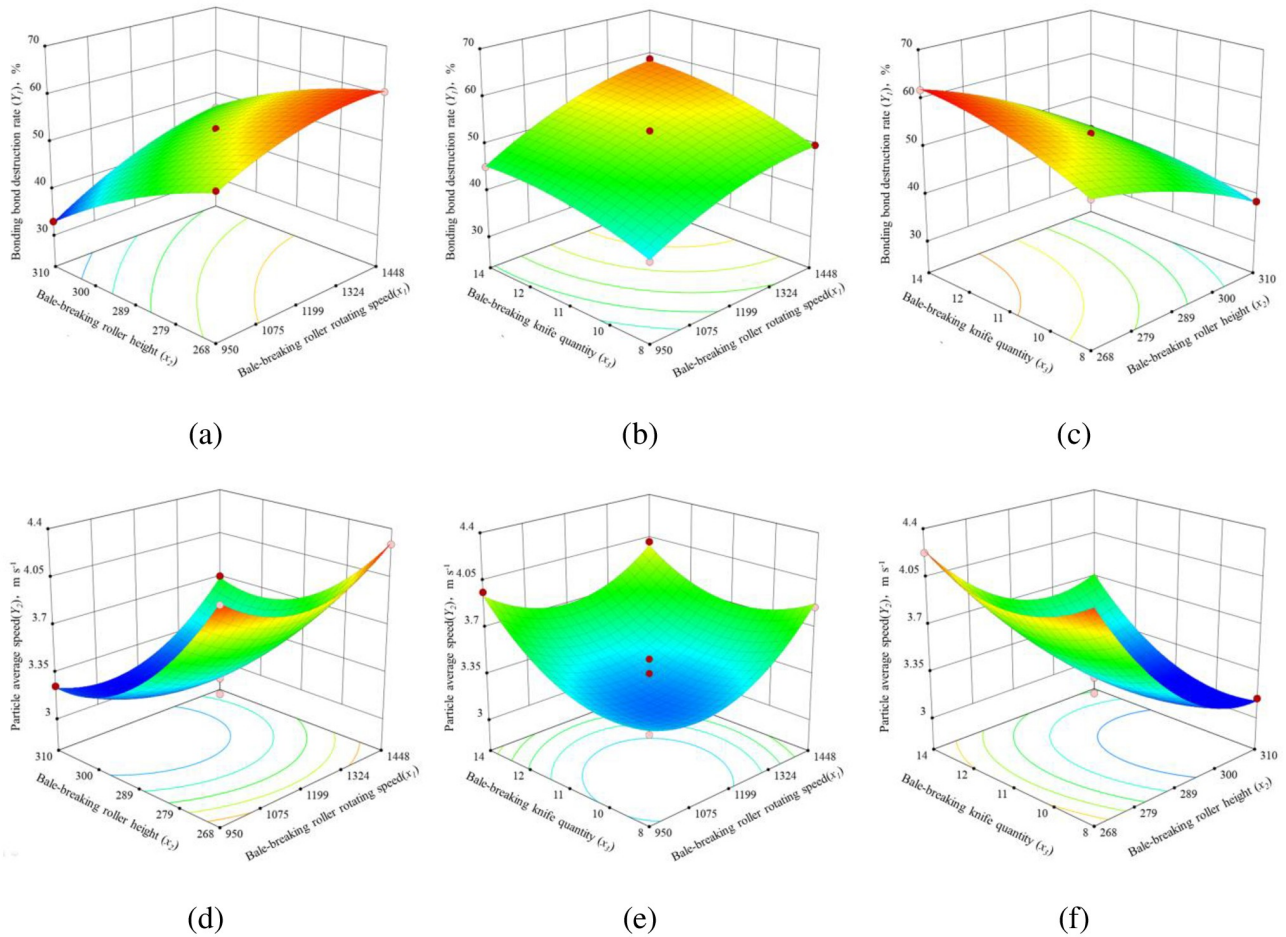
Response surfaces and corresponding contour plots showing the interactions. (a) bale-breaking roller rotating speed (*x*_1_) and bale-breaking roller height (*x*_2_) on bonding bond destruction rate (*Y*_1_), (b) bale-breaking roller rotating speed (*x*_1_) and bale-breaking knife quantity (*x*_3_) on bonding bond destruction rate (*Y*_1_), (c) bale-breaking roller height (*x*_2_) and bale-breaking knife quantity (*x*_3_) on bonding bond destruction rate (*Y*_1_), (d) bale-breaking roller rotating speed (*x*_1_) and bale-breaking roller height (*x*_2_) on particle average speed (*Y*_2_), (e) bale-breaking roller rotating speed (*x*_1_) and bale-breaking knife quantity (*x*_3_) on particle average speed (*Y*_2_), and (f) bale-breaking roller height (*x*_2_) and bale-breaking knife quantity (*x*_3_) on particle average speed (*Y*_2_).

The interactive impact of two factors on the bonding-bond destruction rate and particle average speed were analyzed by maintaining one factor each from bale-breaking roller rotating speed, bale-breaking roller height, and bale-breaking knife quantity. The response surface of bonding-bond destruction rate and particle average speed to the factors is shown in Fig 6.

When the bale-breaking knife quantity was maintained at 11 pieces (Fig 6a and 6d), the bonding-bond destruction rate exhibited an increasing trend, although it subsequently declined with increases in bale-breaking roller rotating speed and bale-breaking roller height and the maximum bonding-bond destruction rate was obtained when the bale-breaking roller rotating speed was 1199 to 1488 rpm and the bale-breaking roller height was 268 to 289 mm. However, the particle average speed initially decreases with increase in the bale-breaking roller rotating speed and bale-breaking roller height, although it tends to increase after reaching the minimum value when the bale-breaking roller rotating speed is 950 to 1199 rpm and bale-breaking roller height is 289 to 310 mm. The response surface exhibited a higher variation rate of bonding-bond destruction rate in the direction of bale-breaking roller rotating speed than bale-breaking roller height, thereby indicating that the effect of bale-breaking roller rotating speed on bonding-bond destruction rate exceeded that of bale-breaking roller height. The variation rate in direction of bale-breaking roller rotating speed exceeded that in the bale-breaking roller height, as shown in the response surface, thereby suggesting a greater impact of bale-breaking roller rotating speed than bale-breaking roller height in determining the particle average speed. This is because the increase of bale-breaking roller rotating speed and bale-breaking roller height increases the amount of straw feeding per unit time, and the interaction between the two increases *Y*_2_ significantly, and has a more significant effect on *Y*_1_.

As shown in Fig 6b and 6e, when the bale-breaking roller height remained at 289 mm, the bonding-bond destruction rate and the particle average speed were positively correlated with bale-breaking roller rotating speed and bale-breaking knife quantity. The response surface implied that particle average speed affected by bale-breaking roller rotating speed and bale-breaking knife quantity was the same. Therefore, the highest bonding-bond destruction rate value was reached when bale-breaking roller rotating speed was within 1199 to 1488 rpm and bale-breaking knife quantity was within 11 to 14 pieces. Thus, the maximum particle average speed was obtained when bale-breaking roller rotating speed coded value from -1 to 1 and bale-breaking knife quantity were 0 to 1, and bale-breaking roller rotating speed were 1199 to 1448 rpm and bale-breaking knife quantity coded value from -1 to 1, respectively. The bonding-bond destruction rate in the response surface was observed as more sensitive to changes in bale-breaking roller rotating speed than bale-breaking knife quantity given that the slope in the direction of bale-breaking roller rotating speed exceeds bale-breaking knife quantity. This is because the higher the bale-breaking roller rotating speed, the more the bale-breaking knife quantity, the lower the feeding limit of the baled-straw, the more the material is fed in the same time, the faster the breakage and discharge efficiency, the higher the quantity and quality of the yarn per unit time, and the improvement of *Y*_1_ and *Y*_2_.

When bale-breaking roller rotating speed was constant at 1199 rpm (Fig 6c and 6f), the bonding-bond destruction rate initially increased with the bale-breaking roller height and bale-breaking knife quantity although it subsequently declined. However, the particle average speed decreased with bale-breaking roller height and bale-breaking knife quantity until the lowest peak was reached and subsequently increased when the two structure parameters continued to increase. The maximum value was estimated when bale-breaking roller height ranged between 268 to 289 mm. And the greatest bonding-bond destruction rate and particle average speed estimated at bale-breaking knife quantity was respectively 11 to 14 pieces and 8 to 14 pieces. The response surface from suggests higher impact of bale-breaking roller height on the bonding-bond destruction rate and particle average speed as opposed to bale-breaking knife quantity. This is because the smaller the bale-breaking roller height, the greater the force of the material in the feeding process, the more significant the sliding cutting effect, the increase in the number of straw per unit time and the improvement of bale breaking quality, promote the improvement of *Y*_1_ and *Y*_2_.

One of the most significant novel contributions of this research is the recognition of the interdependency between these parameters (feed roller speed, feed gap, and knife quantity). Many previous studies have investigated each of these parameters in isolation, but this research establishes how they interact and collectively influence the overall machine performance. For example, while increasing the feed roller speed can increase throughput, it may necessitate adjustments to the feed gap and knife quantity to maintain the quality of the straw treatment. This multivariable optimization approach allows for more precise and adaptable machine settings, which could be particularly beneficial in agricultural operations where the type of straw and operational conditions vary.

### Optimization and verification of regression models

As discussed earlier, bale-breaking rate is one of the most important assessment criteria for a square-bale maize straw breaking. Unbroken-bale maize straw, with high density and low speed, could deteriorate silken quality. Therefore, high bonding-bond destruction rate is required during square-bale maize straw breaking. Meanwhile, the particle average speed is required to be as high as possible. Based on the above analysis, increasing bonding-bond destruction rate will lead to the improvement of bale-breaking rate, resulting in a large amount of maize straw breaking and increase of filamentous rate. To balance the contradicting optimization objectives, a comprehensive evaluation method was applied in a coded range of -1 ≤ *x*_*i*_ ≤ 1 (*i* = 1, 2 and 3). The bonding-bond destruction rate and particle average speed were set as maximize, respectively, and bale-breaking roller rotating speed, bale-breaking roller height and bale-breaking knife quantity were selected in range of -1 to 1 in the experiment level. The optimization objective was then defined as follows:

$$\left\{\begin{array}{l} \text{max}\;Y_{1}\left(x_{1},x_{2},x_{3}\right)\\ \text{max}\;Y_{2}\left(x_{1},x_{2},x_{3}\right)\\ s.t\left\{\begin{array}{l} 950\leq x_{1}\leq 1448\\ 268\leq x_{2}\leq 310\\ 8\leq x_{3}\leq 14 \end{array}\right. \end{array}\right.$$ (13)

The optimal structure parameters of the feeding and breaking system for square-bale maize straw breaking were determined to be -1 for *x*_1_, 1 for *x*_2_, and *x*_*3*_ by solving the regression equation in EDEM. Thus, the bale-breaking roller rotating speed was 1448 rpm, bale-breaking roller height was 268 mm, and bale-breaking knife quantity was 14 pieces. Predicted values of bonding-bond destruction rate and particle average speed were 64.70% and 4.43 m/s, respectively.

To confirm the predicted results, verification experiments using the optimal parameters determined above were further conducted in the same way described previously, and the relevant operating parameter and performance were also determined. In the verification experiment, due to the inability to consider straw bonding-bond destruction and its particle average speed from a microscopic perspective, straw rubbing rate and per unit power productivity were employed as indicators. In the processes of straw breaking and rubbing filament, bonding-bond destruction and breaking are typically positively correlated. A higher bonding-bond destruction rate implies greater bonding-bond destruction, which may lead to an increased rubbing rate, as the bonding-bond between straw particles diminishes, making it easier for external forces to cause straw breakage. If the bonding-bond destruction rate is high, it indicates that the baled-straw fibers are sufficiently loosened during breaking, which usually increases the likelihood of breakage, thereby enhancing the rubbing rate. Consequently, straw rubbing rate is selected to represent straw bonding-bond destruction. The higher the average speed of straw particles, the more straw can typically be processed or transformed within a unit time. This increased speed can enhance per unit power productivity, as more material is handled within the same timeframe. With the increase in particle speed, the required power will also increase accordingly. Therefore, per unit power productivity is selected to characterize straw particle average speed.

**Fig 7:**
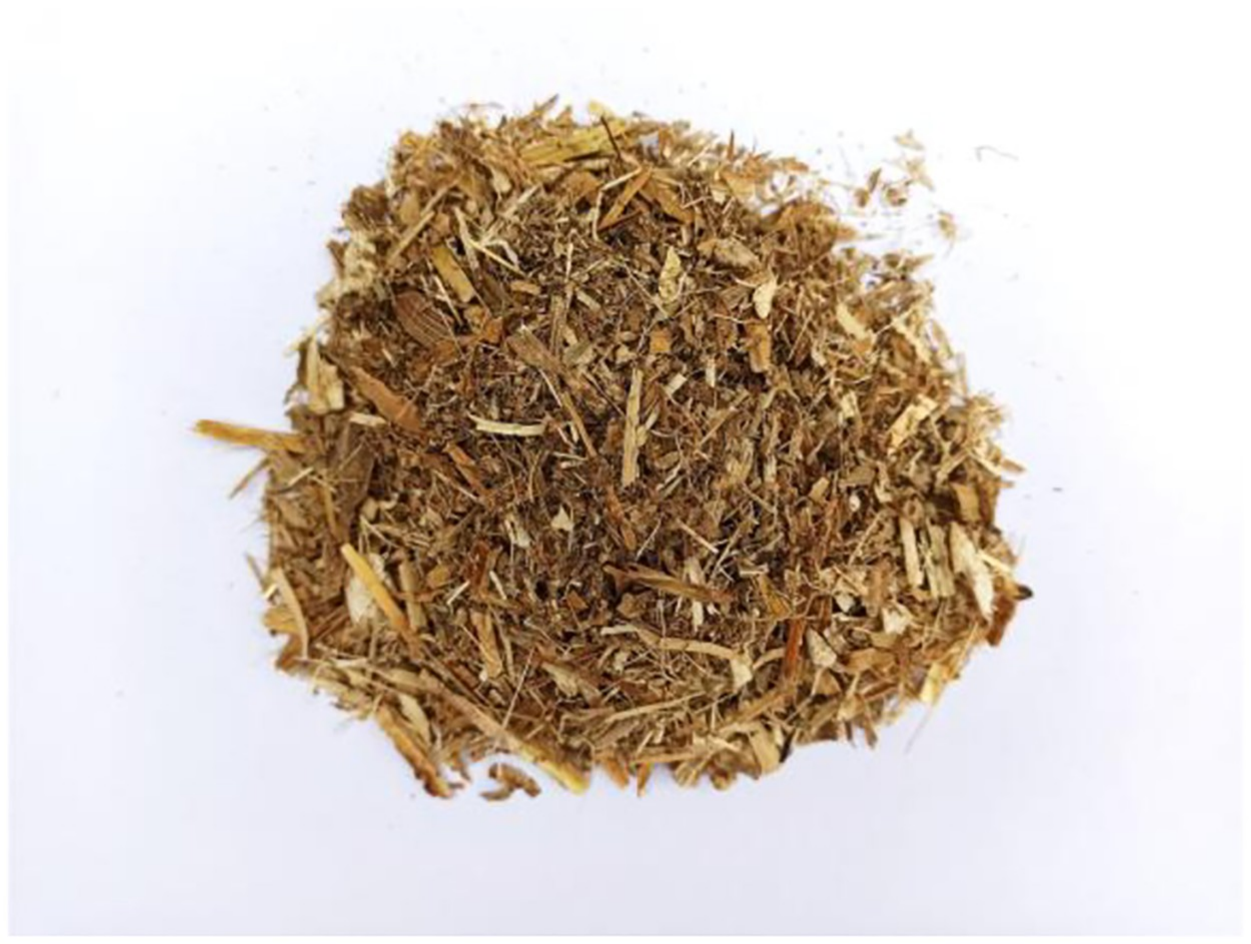
Experiment collected sample of the square-bale maize straw.

As shown in Fig 7, a total of 51 bales of the square-bale maize straw were tested; all the square-bale maize straws were successfully broken. The average value of the straw rubbing rate and per unit power productivity were 96.95% and 235.13 kg/(kW·h), respectively. The results were related to the data obtained from the optimization analysis, indicating that the regression models established in this study were suitable.

## Conclusions

In response to the demand for on-site dispersion treatment of baled straw, a baled-straw feeding and bale-breaking device is proposed. This device integrates an adjustable feeding mechanism that simultaneously performs bale-breaking and feeding processes, thereby could improve the utilization rate of straw localization for small and medium-sized farmers. A square-bale maize straw feeding and bale-breaking simulation model was established based on the optimized results. Simulation results demonstrated that the bale-breaking elements could effectively limit the square-bale movement of the maize straw in the feeding process.

BBD experiment combined with response surface method was employed to optimize square-bale maize straw bale-breaking and rubbing performance. The ANOVA tests demonstrated satisfactory model performances in estimating all parameters with p-values from the goodness of fit tests that are significant at *p* < 0.0100 level and insignificant *p* values from the lack of fit tests (*p* > 0.0500). Additionally, the *R*^2^ for the two models were 0.99 and 0.98. Single and interactive impacts of bale-breaking roller rotating speed (*x*_1_), bale-breaking roller height (*x*_2_), and bale-breaking knife quantity (*x*_3_) on soil stiffness constants were assessed. For the bonding-bond destruction rate modulus the order for interactive factors corresponded to *x*_1_*x*_2_ > *x*_2_*x*_3_ > *x*_1_*x*_3_. For particle average speed modulus the three factors were ordered as *x*_2_*x*_3_ > *x*_1_*x*_3_ > *x*_1_*x*_2_.

The optimal structure parameters were designed to be 1448 rpm for bale-breaking knife roller rotating speed, 268 mm for bale-breaking knife roller height and 14 pieces for bale-breaking knife quantity. Theoretical values of the bonding-bond destruction rate and particle average speed were 64.70% and 4.43 m/s, respectively. Verification experiments using straw rubbing rate and per unit power productivity to represent straw bonding-bond destruction and particle average speed. Results of verification experiment indicate that the simulation results outcomes exhibit a consistent and favorable trend, demonstrating that the device had good bale-breaking performance for baled straw.

The limitations or potential sources of error that may arise during the simulation and optimization process primarily consider the following aspects: On one hand, the model assumes relatively ideal conditions compared to the actual maize straw, and deviations from these assumptions may impact the outcomes of practical production. However, overly complex models may lead to computational instability or exceed computational capacity. Additionally, limitations in boundary conditions during the numerical simulation process may result in errors in the calculation of particle average speed.

This study highlighted the application of dynamic simulation for predicting the kinematic characteristics of baled straw in bale-breaking process. A dynamic analysis gave a relatively accurate prediction of the bonding-bond destruction and particles speed of maize straw. Overall, the adjustable feeding and bale-breaking system developed in this study improved baled straw rubbing performance, and the simulation model has the potential to serve as a simple and reliable prediction method for the development and performance analysis of baled straw adjustable feeding and bale-breaking system.

The novelty of this research lies in its comprehensive and quantified analysis of how the feed roller speed, feed gap, and knife quantity influence the performance of baled straw bale-breaking and rubbing filament machines. By demonstrating the interdependence of these factors, the study introduces a more holistic approach to machine design, offering potential for improved efficiency, energy savings, and straw treatment quality. This breakthrough could ultimately lead to machines that not only optimize operational efficiency but also enhance the sustainability and cost-effectiveness of straw treatment in agricultural systems.

The device proposed in this study requires upgrades and modifications for application in industrial settings. For instance, although the worm gear adjustable feeding mechanism has a self-locking function, it can experience certain deviations in actual operation due to machine vibrations and the working resistance of the bale-breaking roller, resulting in changes in the bale-breaking roller height. Therefore, a locking mechanism should be installed.

In future research, more in-depth and specific studies should be conducted to address technical limitations such as the characteristics of different crop straw and local practical needs. Machine durability: Straw can be abrasive and contains varying levels of moisture, which makes it challenging for rubbing filament equipment to process it consistently over extended periods. Research could focus on improving the durability and lifespan of materials used in the bale-breaking mechanism, including optimizing wear-resistant components. Power consumption: Straw rubbing filament machines require substantial energy to operate effectively, particularly in large-scale applications. Research into more energy-efficient designs could enhance the sustainability of these technologies. Variable straw characteristics: The moisture content, density, and lignin content of straw vary across crops and environmental conditions. Future studies may aim to develop adaptive or customizable equipment to handle different types of straw more efficiently, providing a more tailored approach to rubbing filament processing.
